# Short-term outcomes of robotic versus open hepatectomy among overweight patients with hepatocellular carcinoma: a propensity score-matched study

**DOI:** 10.1186/s12893-023-02058-8

**Published:** 2023-06-07

**Authors:** Zhao-Yi Lin, Xiu-Ping Zhang, Guo-Dong Zhao, Cheng-Gang Li, Zhao-Hai Wang, Rong Liu, Ming-Gen Hu

**Affiliations:** 1 Medical School of Chinese PLA, Beijing, 100853 China; 2 Faculty of Hepato-Pancreato-Biliary Surgery, The First Medical Center of Chinese PLA General Hospital, Beijing, 100853 China; 3 Department of Hepatobiliary, Pancreatic Surgical Oncology, The First Medical Center of Chinese PLA General Hospital, Beijing, 100853 China

**Keywords:** Hepatocellular carcinoma, Overweight, Robotic hepatectomy, Open hepatectomy, Outcome

## Abstract

**Background:**

Robotic hepatectomy (RH) has gradually been accepted as it has overcome some of the limitations of open hepatectomy (OH). This study was to compare short-term outcomes in RH and OH for overweight (preoperative body mass index ≥ 25 kg/m²) patients with hepatocellular carcinoma (HCC).

**Methods:**

Perioperative and postoperative data from these patients who underwent RH or OH between January 2010 and December 2020 were retrospectively analyzed. Propensity score matching (PSM) analysis was performed to determine the impact of RH versus OH on the prognosis of overweight HCC patients.

**Results:**

All 304 overweight HCC patients were included, 172 who were underwent RH, and 132 who were underwent OH. After the 1:1 PSM, there were 104 patients in both RH and OH groups. After PSM, the RH group of patients had a shorter operative time, less estimated blood loss (EBL), a longer total clamping time, a shorter postoperative length of stay (LOS), less chance of surgical site infection and less rates of blood transfusion (all P < 0.05) compared to the OH patients. The differences between operative time, EBL and LOS were more significant in obese patients. RH was found to be an independent protective factor of EBL ≥ 400ml relative to OH in overweight patients for the first time.

**Conclusions:**

RH was safe and feasible in overweight HCC patients. Compared with OH, RH has advantages in terms of operative time, EBL, postoperative LOS, and surgical site infection. Carefully selected overweight patients should be considered for RH.

## Introduction

The incidence of hepatocellular carcinoma (HCC) is gradually increasing and it is now the sixth most common cancer and the third leading cause of cancer-related deaths worldwide [1]. Hepatitis B infection is a major risk factor for HCC in Asia and Africa. However, with the worldwide increase in the incidence of metabolic syndrome, non-alcoholic fatty liver disease/non-alcoholic steatohepatitis (NAFLD/NASH) is emerging as a leading cause of HCC [2–5].

With the improvement of living standards over the past decades, metabolic syndrome has become common in developed countries, where obese (BMI ≥ 30 kg/m²) or overweight (30 > BMI ≥ 25 kg/m²) adults constitute a large proportion of population [6, 7]. Patients with metabolic syndrome are at high risk of developing NAFLD/NASH, liver cirrhosis, liver failure, and HCC [8]. Surgical resection is recommended as the first-line curative treatment for HCC among selected patients [9]. Appropriate surgical decision-making can reduce postoperative complications, shorten hospital stay, and decrease medical expenses. Therefore, it can improve patient rehabilitation and long-term survival. However, surgical risks and severe postoperative complications are more common among overweight patients with HCC [10]. It is therefore essential and urgent to identify risk factors in patients with HCC and high BMI and to improve surgical decision-making and postoperative outcomes.

Robotic surgery has revolutionized the landscape of surgery over the past decade. Robotic hepatectomy (RH) has gradually been accepted as it has overcome some limitations of open hepatectomy (OH), with acceptable postoperative and oncological outcomes [11–14]. Compared with OH, RH has many potential advantages, such as efficient articulation with an almost 540° range of motion, elimination of tremors, and binocular-enhanced 3D vision [15, 16]. Particularly, the risks of surgery increase in overweight patients with thick subcutaneous fat or complex anatomy around the tumor. However, the safety of RH is not clear among overweight patients.

This study aimed to assess the safety and feasibility of RH compared to OH in overweight patients. Our findings can help surgical decision-making to improve the short-term and long-term prognosis of overweight HCC patients.

## Methods

### Patients

A retrospective study was conducted on overweight patients with HCC who underwent curative-intent liver resection between January 2010 and December 2020 at the Chinese People’s Liberation Army (PLA) General Hospital in Beijing. According to the World Health Organization (WHO) classification, BMI was calculated by the following formula: body weight (kg)/height² (m²). BMI was measured within one week before surgery. Patients with 30 kg/m²>BMI ≥ 25 kg/m² were categorized as overweight patients [17, 18]. Patients with BMI ≥ 30 kg/m² were classified as obese patients. As patient identities were anonymized, the informed consent was waived by the Ethics Committee. This study was approved by the Ethics Committee of the PLA general hospital. All RH were performed using the Da Vinci Si Surgical System (Intuitive Surgical, Sunnyvale, CA, USA) by an expert surgical team. The surgical techniques for RH, including the location of trocars, have been reported previously [19, 20].

The inclusion criteria were as follows: (1) BMI ≥ 25 kg/m² with histopathological confirmation of HCC; (2) Sufficient liver function with a Child-Pugh score of ≤ 7; (3) R0 resection as an initial treatment after the learning curve of open or robotic hepatectomy, with no gross or histological sign of HCC in resected specimens; (4) No contraindications for anesthesia or surgery. The exclusion criteria were as follows: (1) A history of other malignant tumors, distant metastases, and preoperative anti-tumor treatment; (2) Absolute contraindications for surgery; (3) Missing data or loss of follow-up.

### Preoperative assessment and postoperative surveillance

We collected the baseline characteristics of the patients, including demographic indicators, preoperative imaging examination and examination results, past medical history, and clinicopathologic characteristics. Clinicopathologic characteristics included the presence of cirrhosis, Child-Pugh grade, maximum tumor size, tumor number, and microvascular invasion.

Postoperative surveillance included quantitative data about surgery, postoperative complications, postoperative examination results, clinicopathologic characteristics, and disease prognosis [21]. The surgical plans were similar in both robotic and open hepatectomy. All patients were informed about the advantages and disadvantages of RH or OH. They voluntarily chose the surgical method after consultation with our team. Postoperative complications were graded according to the Clavien–Dindo classification. All unwanted events in the operating room from preparation for anesthesia were recorded as complications [22]. Cardiac events included acute coronary syndrome, cardiac arrest, and stroke. Respiratory events included respiratory insufficiency requiring invasive ventilation, and pneumonia. Surgical site infection was defined as abdominal effusion with gas in CT scan or ultrasonography in the presence of fever and leukocytosis. Surgical complications included ascites, liver failure, hepatic insufficiency, surgical site infection, bile leakage, and intra-abdominal hemorrhage. Mortality was defined as death within 90 days after surgery. Data were evaluated in January 2022.

### Statistical analysis

All statistical analyses were performed using SPSS v22.0 (SPSS Inc., Chicago, Illinois, USA). Continuous variables with normal distribution are expressed as mean ± standard deviation or median. Categorical variables are expressed as number (n) or proportion (%). Variables with normal distribution were tested by the student’s t-test, whereas variables without normal distribution were tested by the Mann-Whitney U test. A 1:1 propensity score matching (PSM) was performed using the nearest-neighbor matching method to minimize the differences in baseline characteristics between the RH and OH groups. Categorical variables were analyzed using the Chi-squared test or Fisher’s exact test. A P value of < 0.05 was considered statistically significant.

## Results

### Patient characteristics

**Fig. 1:**
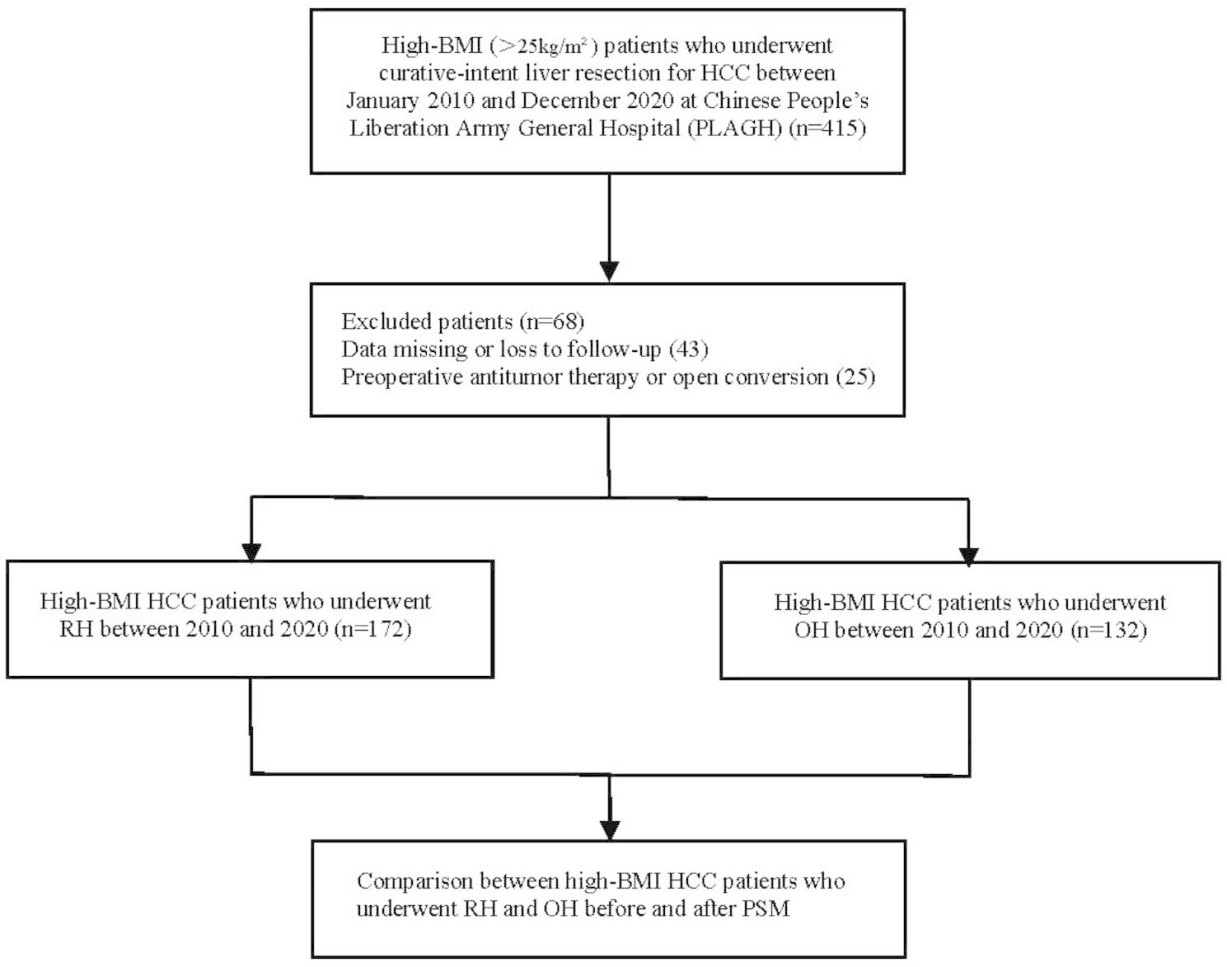
Flow chart of this study showing the selection process of high-BMI HCC patients who underwent RH and OH. (HCC, hepatocellular carcinoma; PSM, propensity score matching; RH, robotic hepatectomy; OH, open hepatectomy, high-BMI, preoperative body mass index ≥ 25 kg/m²)

The flowchart in Fig. 1 shows how the patients were selected for this study. In total, 304 overweight patients with HCC underwent RH or OH between January 2010 and December 2020. Among them, 172 patients were allocated to the RH group and 132 patients were allocated to the OH group. After PSM, the RH and OH groups were matched 1:1, with 104 patients in each group. There was no significant difference between the two groups. The demographics and outcomes of the OH and RH groups before and after PSM are summarized in Table 1.

**Table 1:** The baseline characteristics of high-BMI HCC patients undergoing RH or OH before and after PSM

Variable	Before PSM				After PSM		
	RH group (n = 172)	OH group (n = 132)	*P* value		RH group (n = 104)	OH group (n = 104)	*P* value
Age, years							
< 75	167 (97.1)	129 (97.7)	0.732		101 (97.1)	101 (97.1)	1.000
≥ 75	5(2.9)	3 (2.3)			3 (2.9)	3 (2.9)	
BMI, kg/m²			0.664				0.286
25 ≤ BMI < 30	151 (87.8)	118 (89.4)			89 (85.6)	94 (90.4)	
BMI ≥ 30	21 (12.2)	14 (10.6)			15 (14.4)	10 (9.6)	
Sex							
Female	23(14.0)	23 (17.4)	0.407		15 (14.4)	18 (17.3)	0.569
Male	148 (86.0)	109(82.6)			89 (85.6)	86 (82.7)	
HCC etiology							
HBV	120 (69.8)	98 (74.2)	0.144		72 (69.2)	77 (74.0)	0.496
HCV	5 (2.9)	9 (6.8)			3 (2.9)	5 (4.8)	
HBV and HCV	1 (0.6)	0 (0.0)			1 (1.0)	0 (0.0)	
Others	46 (26.7)	25 (18.9)			28 (26.9)	22 (21.2)	
Diabetes							
No	112 (84.8)	140 (81.4)	0.428		90 (86.5)	90 (86.5)	1.000
Yes	20 (15.2)	32 (18.6)			14 (13.5)	14 (13.5)	
AFP, ng/mL							
≤ 400	137 (79.7)	96 (72.7)	0.157		80 (76.9)	74 (71.2)	0.343
> 400	35 (20.3)	36 (27.3)			24 (23.1)	30 (28.8)	
ALB, g/L							
< 35	14 (8.1)	14 (10.6)	0.461		8 (7.7)	11 (10.6)	0.470
≥ 35	158 (91.9)	118 (89.4)			96 (92.3)	93 (89.4)	
ALT, U/L							
≤ 44	136 (79.1)	96 (72.7)	0.149		75 (72.1)	78 (75.0)	0.637
> 44	36 (20.9)	36 (27.3)			29 (27.9)	26 (25.0)	
TBIL, mol/L							
≤ 17	121 (70.3)	96 (72.7)	0.649		75 (72.1)	77 (74.0)	0.755
> 17	51 (29.7)	36 (27.3)			29 (27.9)	27 (26.0)	
PT, seconds							
≤ 13	24 (14.0)	31 (23.5)	**0.032**		21 (20.2)	21 (20.2)	1.000
> 13	148 (86.0)	101 (76.5)			83 (79.8)	83 (79.8)	
PLT, 10^9^/L							
< 100	15 (8.7)	15 (11.4)	0.444		9 (8.7)	10 (9.6)	0.810
≥ 100	157 (91.3)	117 (79.3)			95 (91.3)	94 (90.4)	
Child-Pugh grade							
A	167 (97.1)	122 (92.4)	0.062		99 (95.2)	102 (98.1)	0.249
B7	5 (2.9)	10 (7.6)			5 (4.8)	2 (1.9)	
Cirrhosis							
No	112 (65.1)	105 (79.5)	**0.006**		79 (76.0)	82 (78.8)	0.619
Yes	60 (34.9)	27 (20.5)			25 (24.0)	22 (21.2)	
No. of tumors							
Solitary	160 (93.0)	124 (93.9)	0.749		96 (92.3)	97 (93.3)	0.789
Multiple	12(7.0)	8(6.1)			8 (7.7)	7 (6.7)	
Tumor diameter, cm							
≤ 5	117 (68.0)	67 (50.8)	**0.002**		60 (57.7)	55 (52.9)	0.486
> 5	55 (32.0)	65 (49.2)			44 (42.3)	49 (47.1)	
Microvascular invasion							
Absent	155 (90.1)	102 (77.3)	**0.002**		90 (83.2)	83 (79.6)	0.194
Present	17 (9.9)	30 (22.7)			14 (13.5)	21 (20.2)	
Tumor encapsulation							
Complete	138 (80.2)	83 (62.9)	**0.001**		76 (73.1)	72 (69.2)	0.540
Incomplete or absent	34 (19.8)	49 (37.1)			28 (26.9)	32 (30.8)	

Data are presented as n (%). Bold text hinted that these variables were statistically significant

HCC, hepatocellular carcinoma, PSM, propensity score matching; RH, robotic hepatectomy; OH, open hepatectomy; ASA, American Society of Anesthesiologists; HBV, hepatitis B virus; HCV, hepatitis C virus; AFP, α-fetoprotein; ALB, albumin; ALT, alanine aminotransferase; TBIL, total bilirubin; PT, prothrombin time; PLT, platelet.

### Perioperative outcomes of overweight patients with HCC undergoing RH or OH before and after PSM

The perioperative outcomes are summarized in Table 2. After PSM, patients in the RH group had shorter operation time (median 170 vs. 184.5 min, P = 0.048), less estimated blood loss (EBL) (median 75.0 vs. 300.0 mL, P < 0.001), and less blood transfusion (9.6% vs. 19.2%, P = 0.048) compared to those in the OH group. Although the incidence of Pringle manipulation was similar between groups (78.8% vs. 84.6%, P = 0.282), the clamping time (median 25.5 vs. 18.0, P = 0.041) was longer in the RH group than in the OH group. After PSM, there were significant differences in minor surgical complications (Clavien-Dindo I–II) between the groups, including ascites, surgical site infection, and intra-abdominal hemorrhage (P = 0.030). In particular, the RH group had lower surgical site infection (0.0% vs. 3.8%). The RH group also had a significantly less postoperative hospital stay than the OH group after PSM (median 5.0 vs. 9.0 days, P < 0.001). No significant difference was observed in 90-day mortality between the two groups after PSM (P > 0.05).

**Table 2:** The perioperative outcomes of high-BMI HCC patients undergoing RH or OH before and after PSM

Variable	Before PSM				After PSM		
	RH group (n = 172)	OH group (n = 132)	*P* value		RH group (n = 104)	OH group (n = 104)	*P* value
Operative time, min	147.5 (100.5, 210.0)	190.0 (153.5, 233.75)	**< 0.001**		170.0 (110.5, 225.8)	184.5 (155.0,226. 3)	**0.048**
Estimated blood loss, ml	50.0 (50.0, 200.0)	300.0 (162.5, 600.0)	**< 0.001**		75.0 (50.0, 200.0)	300.0 (100.0, 600.0)	**< 0.001**
Blood transfusion							
Yes	13 (7.6)	26 (19.7)	**0.002**		10 (9.6)	20 (19.2)	**0.048**
No	159 (92.4)	106 (80.3)			94 (90.4)	84 (80.8)	
Pringle maneuver							
Yes	134 (77.9)	111 (84.1)	0.177		82 (78.8)	88 (84.6)	0.282
No	38 (22.1)	21 (15.9)			22 (21.2)	16 (15.4)	
Total clamping time, min	24.5 (12.3, 39.8)	21.0 (10.3, 33.0)	0.261		25.5 (14.3, 44.0)	18.0 (10.25, 32.0)	**0.041**
Postoperative AFP, ng/mL							
≤ 400	169 (98.3)	126 (95.5)	0.153		102 (98.1)	98 (94.2)	0.149
> 400	3 (1.7)	6 (4.5)			2 (1.9)	6 (5.8)	
Nonoperative complications							
Yes	5 (2.9)	7 (5.3)	0.288		3 (2.9)	5 (4.8)	0.471
NO	167 (97.1)	125 (94.7)			101 (97.1)	99 (95.2)	
Type of nonoperative complications ¶							
Cardiac events	3 (1.7)	3 (2.3)	0.479		2 (1.9)	2 (1.9)	0.600
Respiratory events	2 (1.2)	4 (3.0)			1 (1.0)	3 (2.9)	
Minor surgical complications (Clavien-Dindo I–II)							
Yes	2 (1.2)	12 (9.1)	**0.001**		2 (1.9)	9 (8.7)	**0.030**
No	170 (98.8)	121 (90.9)			102 (98.1)	95 (91.3)	
Major surgical complications (Clavien-Dindo III–V)							
Yes	2 (1.2)	4 (3.0)	0.246		2 (1.9)	2 (1.9)	1.000
No	170 (98.8)	127 (97.0)			102 (98.1)	102 (98.1)	
Type of surgical complications ¶							
Liver failure	0 (0.0)	2 (1.5)	**0.041**		0 (0.0)	0 (0.0)	0.348
Hepatic insufficiency	1 (0.6)	1 (0.8)			1 (1.0)	1 (1.0)	
Bile leakage	1 (0.6)	1 (0.8)			1 (1.0)	1 (1.0)	
Surgical site infection	0 (0.0)	5 (3.8)			0 (0.0)	4 (3.8)	
Ascites	1 (0.6)	4 (3.0)			1 (1.0)	3 (2.9)	
Intra-abdominal hemorrhage	1 (0.6)	2 (1.5)			1 (1.0)	2 (1.9)	
Others	0 (0.0)	1 (0.8)			0 (0.0)	0 (0.0)	
Postoperative LOS, day	5.0 (4.0, 7.0)	9.0 (7.0, 11.0)	**< 0.001**		5.0 (4.0, 7.0)	9.0 (7.0, 11.0)	**< 0.001**
90-day mortality	1 (0.6)	1 (0.8)	0.851		1 (1.0)	0 (0.0)	0.316

Data are presented as n (%) or median (IQR), Bold text hinted that these variables were statistically significant. ¶, duplications present

HCC, hepatocellular carcinoma, PSM, propensity score matching; RH, robotic hepatectomy; OH, open hepatectomy; LOS, length of stay.

### Subgroup analysis for perioperative outcomes of obese patients with HCC undergoing RH or OH

There were 35 obese patients with HCC after PSM, including 21 patients in the RH group and 14 patients in the OH group. Their perioperative outcomes are summarized in Table 3. The RH subgroup of patients had shorter operative time (median 135 vs. 204 min, P = 0.005), less EBL (median 50.0 vs. 350.0 mL, P < 0.001), and less postoperative hospital stay (median 4.0 vs. 9.0, P < 0.001) compared to the OH patients. These differences were greater than those observed among overweight patients.

**Table 3:** The perioperative outcomes of obese HCC patients undergoing RH or OH

Variable	RH group (n = 21)	OH group (n = 14)	*P* value
Operative time, min	135.0 (94.5, 198.5)	204.5 (187.5, 236.5)	**0.005**
Estimated blood loss, ml	50.0 (50.0, 100.0)	250.0(187.5, 525.0)	**< 0.001**
Blood transfusion			
Yes	0 (0.0)	2 (14.3)	0.074
No	21 (100.0)	12 (85.7)	
Pringle maneuver			
Yes	15 (71.4)	13 (92.9)	0.121
No	6 (28.6)	1 (7.1)	
Total clamping time, min	20.0 (0.0, 33.0)	28.0 (14.3, 47.0)	0.171
Postoperative AFP > 400 ng/mL	1 (4.8)	0 (0.0)	
Nonoperative complications			
YES	0 (0.0)	2 (14.3)	0.074
No	21 (100.0)	12 (85.7)	
Type of nonoperative complications ¶			
Cardiac events	0 (0.0)	1 (7.1)	0.204
Respiratory events	0 (0.0)	1 (7.1)	
Complications			
Yes	1 (4.8)	2 (14.3)	0.324
No	20 (95.2)	12 (85.7)	
Type of surgical complications ¶			
Ascites	1 (4.8)	0 (0.0)	0.153
Liver failure	0 (0.0)	0 (0.0)	
Hepatic insufficiency	0 (0.0)	0 (0.0)	
Surgical site infection	0 (0.0)	2 (14.3)	
Bile Leakage	0 (0.0)	0 (0.0)	
Intra-abdominal hemorrhage	0 (0.0)	0 (0.0)	
Others	0 (0.0)	0 (0.0)	
Postoperative LOS, day	4.0 (3.0, 7.0)	9.0 (8.0, 11.3)	**< 0.001**
90-day mortality	0 (0.0)	0 (0.0)	1.000

Data are presented as n (%) or median (IQR), Bold text hinted that these variables were statistically significant. **Abbreviation**: HCC, hepatocellular carcinoma; RH, robotic hepatectomy; OH, open hepatectomy; LOS, length of stay

### Univariable and multivariable analyses of risk factors associated with EBL ≥ 400 mL in patients with HCC and high BMI undergoing RH or OH

All univariable and multivariable analyses of risk factors associated with EBL ≥ 400mL in patients with HCC and high BMI undergoing RH or OH after PSM are shown in Table 4. Univariable analysis identified surgical approaches, preoperative ALB, maximum tumor diameter, and operation time as independent risk factors of EBL (P < 0.05). These four parameters were included in multivariate analysis. Multivariate analysis revealed that longer operation time (3.692, 95% CI 1.748–7.797, P < 0.001) and larger diameter of tumor (2.422, 95% CI 1.182–4.961, P = 0.016) were independent risk factors for EBL. However, higher preoperative ALB levels (0.237, 95% CI 0.073–0.768, P = 0.016) and RH (compared to OH) (0.133, 95% CI 0.061–0.292, P < 0.001) were protective factors for EBL.

**Table 4:** Univariable and multivariable analyses of risk factors associated with EBL ≥ 400ml in high-BMI HCC patients undergoing RH or OH after PSM

characteristics		Univariate analysis				Multivariate analysis		
		B	HR (95%CI)	*P* value		B	HR (95%CI)	*P* value
RH vs. OH	-1.792	0.167 (0.083–0.335)	**< 0.001**		-2.014	0.133 (0.061–0.292)	**< 0.001**	
Age ≥ 75	0.748	2.113 (0.242–18.469)	0.499					
Male	-0.120	1.919 (0.934–2.038)	0.778					
HBV	0.652	4.078 (2.111–3.944)	0.076					
Diabetes	-0.042	0.958 (0.397–2.312)	0.925					
AFP > 400ng/mL	0.595	1.814 (0.942–3.492)	0.075					
ALB ≥ 35 g/L	-1.589	0.204 (0.076–0.548)	**0.002**		-1.439	0.237 (0.073–0.768)	**0.016**	
ALT > 44 U/L	0.103	1.108 (0.567–2.167)	0.764					
TBIL > 17 mol/L	-0.423	0.655 (0.323–1.330)	0.242					
PT > 13 s	0.046	1.047 (0.496–2.212)	0.904					
PLT ≥ 100 × 10^9^/L	-0.117	0.889 (0.322–2.459)	0.821					
Child-Pugh grade B7	0.615	1.849 (0.401–8.520)	0.430					
Cirrhosis	0.286	1.330 (0.664–2.665)	0.420					
Multiple tumors	-0.142	0.868 (0.265–2.839)	0.814					
Tumor diameter > 5 cm	1.017	2.764 (1.492–5.118)	**0.001**		0.884	2.422 (1.182–4.961)	**0.016**	
Microvascular invasion	0.728	2.071 (0.978–4.382)	0.057					
Tumor encapsulation incomplete	0.266	1.305 (0.684–2.490)	0.420					
Operative time ≥ 180	1.406	4.078 (2.111–7.879)	**< 0.001**		1.306	3.692 (1.748–7.797)	**0.001**	

Bold text hinted that these variables were statistically significant

EBL, estimated blood loss; BMI, body mass index; HCC, hepatocellular carcinoma; RH, robotic hepatectomy; OH, open hepatectomy; CI confidence interval; HBV, hepatitis B virus; AFP, α-fetoprotein; ALB, albumin; ALT, alanine aminotransferase; TBIL, total bilirubin; PT, prothrombin time; PLT, platelet.

## Discussion

The number of overweight patients developing HCC may increase in the future [23], which increases the need for hepatectomy. Previous studies have shown that preoperative high BMI is an independent risk factor for 30-day morbidity and short-term postoperative complications among patients undergoing HCC resection [24]. The surgical method also affects the postoperative outcome of patients [12, 25]. However, there are still no reports on the short-term outcomes of RH compared with OH among overweight patients. Whether overweight patients can also benefit from RH is still unknown.

For the first time, this study compared the short-term outcomes of RH or OH among overweight patients with HCC. In this study, patients undergoing RH experienced a lower rate of minor complications (1.9% vs. 8.7%), shorter duration of surgery (170.0 min vs. 184.0 min), and shorter hospital stay (5.0 d vs. 9.0 d), compared with patients undergoing OH. RH had a unique advantage regarding EBL (75.0 mL vs. 300.0 mL, P < 0.05). Multivariable analyses indicated that the surgical method (RH or OH) was an independent risk factor for EBL ≥ 400 mL.

This large cohort of HCC patients undergoing RH or OH at a tertiary cancer center demonstrated that the robotic approach was superior to the open approach in terms of short-term outcomes, such as intraoperative blood loss, adhesion, bile leakage, and postoperative length of hospital stay [14, 26]. In addition, this study indicated that the short-term outcomes of overweight patients with HCC were similar between the RH and OH groups. These differences are attributed to the advantages of the Da Vinci robotic system, such as improved vision through three-dimensional view, magnification, attenuation of tremors, and flexibility of the instrument, which allows precise manipulation techniques in various surgical procedures [27]. Patients with high BMI usually have more difficult surgeries. Hyperglycemia and immunosuppression may increase the risk of perioperative complications in overweight patients [24]. Compared with OH, RH significantly reduced operation time, intraoperative blood loss, and the need for transfusion in the present study. The flexibility of the robotic arm may be advantageous in the small abdominal space of patients with high BMI. Furthermore, the surgeon’s hand does not blind the visual field in RH, which helps timely detection of intraoperative bleeding and tissue damage. Minimally invasive approaches are associated with significantly reduced perioperative inflammation, which accelerates recovery. [28, 29].

High BMI has been an independent risk factor for postoperative morbidity in many studies [21, 30]. In the present study, compared with RH, OH led to a higher incidence of postoperative minor complications (Clavien-Dindo I–II), particularly surgical site infection. The cooperation between the robotic arm and abdominal lens in RH reduces the length of abdominal wall incision and decreases abdominal wall and peritoneal tissue injury when exposing the surgical site, thereby reducing intraoperative infection. Reduced abdominal infection and trauma resulted in a shorter hospital stay in the RH group in this study. In addition, 30-day mortality and postoperative complications were lower in the RH group, suggesting a short-term advantage of RH. However, future randomized-controlled trials and long-term follow-up are needed.

High BMI has close correlations with diabetes mellitus, NAFLD, and NASH [31]. These complications greatly increase the incidence of postoperative pneumonia, cardiac events, and recovery time. Even, some patients with HCC cannot undergo surgery due to severe obesity. In subgroup analyses, the RH group had significantly less surgical site infection (0.0% vs. 14.3%). Several reasons may explain the above associations. First, robotic surgery allows faster postoperative activity in this subgroup of patients and reduces the chance of intraperitoneal adhesions[32]. Second, the smaller incision reduces the inflammatory response [33]. Finally, adipose tissue has lower nerve endings and blood vessels than other tissues, which can increase the risk of postoperative infection [24]. Although RH effectively increases operational tolerance among obese patients with HCC, indications for a robotic surgery should be carefully evaluated due to the difficulty of surgery in obese patients.

Previous reports elaborately addressed the association between EBL and surgical outcomes in HCC resection [34]. EBL is also closely related to tumor size, tumor vascular invasion, preoperative nutritional status, and duration of operation [35]. Our findings showed that EBL correlates with tumor size, duration of operation, and preoperative albumin levels. For the first time, we found that RH was an independent protective factor for EBL relative to OH in overweight patients with HCC. RH with a clearer surgical field can better detect small intraoperative blood vessels and reduce EBL. Compared to OH, RH can also effectively shorten the duration of surgery. These advantages of RH can effectively reduce EBL.

This study has several limitations. First, this is a retrospective study with its inherent defects, even though PSM was used to reduce selection bias. Second, this study was a single-center study. Although our study had a large sample size, multi-center studies or randomized-controlled trials are still needed. Lastly, this study only enrolled Chinese patients who had mostly HBV-related cirrhosis, and NAFLD-related HCC was less common in this study. This study should be validated in the Europe and US, where NAFLD-related HCC is more common.

In conclusion, for the first time, this study demonstrated that RH is safe and feasible in overweight patients with HCC, and some of its short-term results are better than OH. But more systematic multi-center randomized controlled trials are still needed to be verified in the future. Carefully selected overweight patients should be considered for RH.

## Data Availability

The datasets generated and/or analyzed during the current study are not publicly available, but are available from the corresponding author on reasonable request.
